# Reactivity and sensitivity of commercially available influenza rapid diagnostic tests in Japan

**DOI:** 10.1038/s41598-017-14536-0

**Published:** 2017-11-03

**Authors:** Yuko Sakai-Tagawa, Seiya Yamayoshi, Chiharu Kawakami, Mai Q. Le, Yuko Uchida, Takehiko Saito, Chairul A. Nidom, Ira Humaira, Kathy Toohey-Kurth, Abdel-Satar Arafa, Ming-Tsan Liu, Yuelong Shu, Yoshihiro Kawaoka

**Affiliations:** 1 Division of Virology, Department of Microbiology and Immunology, Institute of Medical Science, University of Tokyo, Tokyo, Japan; 2 Yokohama City Institute of Public Health, Kanagawa, Japan; 3 National Institute of Hygiene and Epidemiology, Quận Hai Bà Trưng, Vietnam; 4 Influenza and Prion Disease Research Center, National Institute of Animal Health, Tsukuba, Japan; 5 AIRC Laboratory, Faculty of Veterinary Medicine, Airlangga University, Surabaya, Indonesia; 6 AIRC Laboratory, School of Medicine, Airlangga University, Surabaya, Indonesia; 7 Wisconsin Veterinary Diagnostic Laboratory, School of Veterinary Medicine, University of Wisconsin-Madison, Madison, USA; 8 National Laboratory for Veterinary Quality Control on Poultry Production, Animal Health Research Institute, Giza, Egypt; 9 Center for Diagnostics and Vaccine Development, Centers for Disease Control, Taipei, Taiwan; 10 National Institute for Viral Disease Control and Prevention, China Centers for Disease Control and Prevention, Beijing, China; 11 Department of Pathobiological Sciences, School of Veterinary Medicine, University of Wisconsin-Madison, Madison, USA; 12 Department of Special Pathogens, International Research Center for Infectious Diseases, Institute of Medical Science, University of Tokyo, Tokyo, Japan; 13 ERATO Infection-Induced Host Responses Project, Japan Science and Technology Agency, Saitama, Japan

## Abstract

Seasonal influenza virus routinely causes epidemic infections throughout the world. Sporadic infections by H5N1, H5N6, and H7N9 viruses are also reported. To treat patients suffering from such viral infections, broadly reactive and highly sensitive influenza rapid diagnostic tests (IRDTs) are required. Here, we examined the reactivity and sensitivity of 25 IRDTs available in Japan for the detection of seasonal H1N1pdm09, H3N2, and type B viruses, as well as highly pathogenic H5 and H7 viruses. All of the IRDTs tested detected the seasonal viruses and H5 and H7 viruses albeit with different sensitivities. Several IRDTs detected the H5 and H7 viruses and the seasonal viruses with similar (high) sensitivity.

## Introduction

Influenza is one of the most prevalent infectious diseases in the world. Seasonal influenza viruses, including H1N1pdm09, H3N2, and type B viruses, are responsible for high morbidity and mortality especially among the elderly and immunocompromised individuals. Despite the availability of influenza vaccines, seasonal influenza viruses cause epidemics every year. Moreover, other subtypes of influenza A virus from other animal species have sporadically transmitted to humans. For example, highly pathogenic avian influenza H5N1 viruses are circulating among poultry in eastern Asia and Egypt and transmit to humans^1^. Reassortant viruses (H5N2, H5N6, and H5N8 viruses) that possess the hemagglutinin (HA) segment of a highly pathogenic avian H5N1 virus and the neuraminidase (NA) segment of another subtype have emerged because of the sustained circulation of highly pathogenic avian H5N1 viruses among birds. H5N6 viruses also cause sporadic infection in humans^2^, and H5N2 virus replicates well in mammalian hosts^3,4^. In addition to these H5 viruses, human infections with avian influenza H7N9 virus were first reported in 2013^5^. Since then, the H7N9 virus has infected humans every influenza season, with the fifth wave occurring in the 2016‒17 season^6^. During the fifth wave, highly pathogenic H7N9 viruses possessing HA with multi-basic amino acids at the cleavage site were isolated from avian and human cases^7,8^. It is difficult to prepare vaccines against these viruses in a timely manner. Therefore, the first line of defense against H5 and H7 virus infections is antiviral drugs, such as NA inhibitors.

For optimum efficacy, the NA inhibitors (oseltamivir, zanamivir, peramivir, and laninamivir) should be administered within 2 days of symptom onset^9,10^. Healthcare providers therefore need a rapid, easy, and sensitive diagnosis test. For influenza diagnosis, basic virologic approaches such as virus isolation and RT-PCR have been used, but these methods require time and specialized techniques, so they are not suitable in the clinical setting. To overcome this constraint, influenza rapid diagnostic tests (IRDTs) have been developed and are now widely used even at the local, small clinic level in Japan. However, conventional IRDTs fail to detect influenza viruses at early time points after onset^11,12^. Recently, some manufacturers developed analyzers to increase the sensitivity of IRDTs. These analyzers are able to evaluate the results instead of relying on the human eye. Here, we examined the sensitivity of 25 IRDTs (4 IRDTs that used analyzers and 21 conventional IRDTs) for various isolates of seasonal influenza A and B viruses as well as for human and avian H5 and H7 viruses, which possess the potential to transmit to humans^13^.

## Results and Discussion

We evaluated the sensitivity of 25 IRDTs commercially available in Japan in 2017 (Table 1). These IRDTs are optimized to detect seasonal influenza, including H1N1pdm09, H3N2, and type B viruses, by using mouse monoclonal antibodies against the influenza A and B virus nucleoproteins (NPs), which are conserved among the influenza A or B viruses. Because the epitopes on NP are conserved among type A viruses, it is stated that 20 of the 25 IRDTs (the exceptions being QuickNavi Flu, QuickNavi-Flu+RSV, Nanotrap Flu A•B, BD Veritor System Flu, and Rapiim Flu-AB) can detect several avian influenza A viruses, across subtypes H1 through H15. The major determinant of the sensitivity of the IRDTs is the reactivity of the monoclonal antibody against the NP used in the IRDT. In addition, the composition of the lysis buffer, the proportion of sample in the analyte, and the method used to visualize the results can affect the sensitivity. The 25 IRDTs can be divided into two formats: the test strip format and the well format. The well format can be further subdivided into two groups based on how the result is evaluated: BD Veritor System Flu, Fuji dri-chem immuno AG cartridge FluAB, Spotchem FLORA FluAB, and Rapiim Flu-AB require a specific analyzer to evaluate the results, whereas the other well format types are assessed by the human eye. These analyzers can only read one sample at a time; although BD Veritor System Flu and Spotchem FLORA FluAB require less than one minute to read, Fuji dri-chem immuno AG cartridge FluAB and Rapiim Flu-AB require 10‒15 min and 7.5 minutes, respectively. Therefore, patients wait times for results are extended when many influenza patients come to a clinic that has only one analyzer. In contrast, human eye-judged IRDTs can be used to test many samples in parallel. Mechanistically, 23 of the IRDTs employ an immunochromatographic method, whereas Immunotrap Influenza A•B utilizes magnetic energy for the movement of the immune-complexes, and Rapiim Flu-AB detects the immune-complexes by light scattering. All 25 IRDTs take between 1 and 15 min to complete each test.

**Table 1:** Influenza rapid diagnosis tests (IRDTs) evaluated in this study.

IRDT	Manufacturer	Format^a^	Input ratio^b^ (%)	Minutes to assess^c^
Statmark FLU Stick-N	Nichirei Biosciences	Test strip	100	1–10
RapidTesta color FLU stick	Sekisui Medical	Test strip	100	2–10
QuickVue Rapid SP influ	DS Pharma Biomedical	Test strip	100	10
Clearview Exact Influenza A&B	Alere Medical	Test strip	100	8
Espline Influenza A&B-N	Fujirebio	Well	6.7	15
ImmunoAce Flu	Tauns Laboratories	Well	12.5	3–8
Brightpoc Flu	Nichirei Biosciences	Well	13.8	1–10
Immunofine FLU	Nichirei Biosciences	Well	3.8	1–10
Spotchem i-Line FluAB-S	Arkray-factory	Well	8.75	1–10
QuickNavi Flu	Denka Seiken	Well	10	3–8
QuickNavi-Flu+RSV	Denka Seiken	Well	10	3–8
Goldsign FLU	Institute of Immunology	Well	14.5	1–8
Prorast Flu One	Adtec	Well	16.7	8
Finevision Influenza	Alere Medical	Well	10	5
RapidTesta FLU•NEO	Sekisui Medical	Well	19.2	15
Nanotrap Flu A•B	ROHTO Pharmaceutical	Well	8.3	3–8
Quick Chaser Flu A,B (Type H)	Mizuho Medy	Well	25	5–10
Alsonic Flu	Alfresa pharma	Well	10	5
Primecheck Flu (Type S)	Alfresa pharma	Well	20.8	3–10
Primecheck Flu•RSV	Alfresa pharma	Well	13	5–10
BD Veritor System Flu	Becton Dickinson	Well + Analyzer	13.3	5–10
Fuji dri-chem immuno AG cartridge FluAB	Mizuho Medy	Well + Analyzer	21.4	3–15
Spotchem FLORA FluAB	Arkray-factory	Well + Analyzer	13.6	1.5–10
Immunotrap Influenza A•B	Wako Pure Chemical Industries	Well	5	1
Rapiim Flu-AB	Toshiba medical systems	Well + Analyzer	40	7.5

^a^All IRDTs examined were divided into two types based on their format: (i) test strip format, in which a test strip is dipped into the lysed sample and the reaction occurs on the strip; or (ii) well format, in which the lysed sample is dropped into the well and the reaction occurs inside a covered plastic body. “+Analyzer” means that these IRDTs need an analyzer to evaluate the result. ^b^For all tested IRDTs, the test samples (100 μl) were mixed with lysis buffer (A). All or part of the lysed sample (B) was subjected to the assay. Input ratios were calculated using the following formula: volume B/(100 μl + volume A) × 100. ^c^The time required to obtain the results is based on the individual manufacturer’s instructions.

We examined the sensitivity of each IRDT for influenza viruses of various subtypes isolated between 2013 and 2017 (see Table 2). The detection limit for seasonal influenza A viruses, such as H1N1pdm09 and H3N2 viruses, of the tested IRDTs ranged from 10^2.5^ to 10^6^ TCID_50_ per 100 μl (Table 3). The sensitivity for H1N1pdm09 viruses tended to be higher than that for H3N2 viruses. A similar trend was observed in our previous report^14^. The most sensitive IRDT for seasonal H1N1pdm09 and H3N2 viruses was Prorast Flu One. The detection limit for influenza B viruses, including both lineages, of the tested IRDTs ranged from 10^3^ to 10^6^ TCID_50_ per 100 μl. All tested IRDTs possessed similar or reduced sensitivity for influenza B viruses compared with that for seasonal influenza A viruses (H1N1pdm09 and H3N2 viruses) (Table 3). The most sensitive IRDT for seasonal type B viruses was Fuji dri-chem immuno AG cartridge FluAB.

**Table 2:** Influenza virus isolates used in this study.

Classification	Virus strain	Abbreviation^b^
Seasonal H1N1pdm09	A/Tokyo/UT-IMS1/2014	H1-1
	A/Yokohama/90/2015	H1-2
	A/Tokyo/HP013/2016	H1-3
Seasonal H3N2	A/Tokyo/UT-IMS2-1/2014	H3-1
	A/Tokyo/IMS4-1/2015	H3-2
	A/Tokyo/HP022/2016	H3-3
Seaonal B/Victoria	B/Kamakura/29/2013	B/Vic-1
	B/Kamakura/8/2014	B/Vic-2
	B/Tokyo/HP009/2016	B/Vic-3
Seasonal B/Yamagata	B/Kamakura/1/2013	B/Yam-1
	B/Kamakura/10/2014	B/Yam-2
	B/Tokyo/HP005/2016	B/Yam-3
H5 viruses	A/duck/Vietnam/QT1726-2/2013 (H5N1)^a^	H5-1
	A/duck/Cairo/157CA/2015 (H5N1)^a^	H5-2
	A/chicken/East Java/UT64/2016 (H5N1)^a^	H5-3
	A/bird/Wisconsin/WDSL1-4/2015 (H5N2)^a^	H5-4
	A/muscovy duck/Vietnam/HN1701/2014 (H5N6)^a^	H5-5
	A/muscovy duck/Aomori/1-3 T/2016 (H5N6)^a^	H5-6
	A/chicken/Niigata/1-1 T/2016 (H5N6)^a^	H5-7
H7 viruses	A/Anhui/1/2013 (H7N9)	H7-1
	A/Taiwan/1/2017 (H7N9)^a^	H7-2
	A/Guangdong/17SF003/2016 (H7N9)^a^	H7-3
	A/feline/New York/WVDL-14/2016 (H7N2)	H7-4

^a^Viruses possess HA with multi-basic amino acids at the cleavage site. ^b^Abbreviations used in Tables 3 and 4 are shown.

**Table 3:** Sensitivity of IRDTs for seasonal influenza A and B viruses^a^.

IRDT	Minimum Virus Titer (log_10_ TCID_50_/100 μl) for a positive result with											
	H1-1	H1-2	H1-3	H3-1	H3-2	H3-3	B/Vic-1	B/Vic-2	B/Vic-3	B/Yam-1	B/Yam-2	B/Yam-3
Statmark FLU Stick-N	5	5	5	4	5	5	4	5.5	5	5	5	5
RapidTesta color FLU stick	4	4	4	4	4	5	3	5	4	4.5	4	4
QuickVue Rapid SP influ	4	4	4	4	5	5	4	5.5	5	5	5	4.5
Clearview Exact Influenza A&B	4	4	4	4	4.5	5	3	4	3	4	3	3
Espline Influenza A&B-N	3	3	3.5	3	4	4	3	5	4	4	4	4
ImmunoAce Flu	3	3	3	3	4	4	3	4	4	4	4	4
Brightpoc Flu	4	5	5	4	5	5	4	6	5	5	3	5
Immunofine FLU	4	4	4	4	5	5	4	5	5	5	4	4
Spotchem i-Line FluAB-S	4	4	4	4	5	5	4	6	5	6	3	5
QuickNavi Flu	4	4	4	4	5	5	4.5	6	5	5	3	5
QuickNavi-Flu+RSV	4	4	4	4	5	5	5	6	6	6	3	5
Goldsign FLU	4	4	4.5	4	5	5	5	6	5	5.5	3	5
Prorast Flu One	2.5	3	2.5	3	3.5	4	4	5	5	5	4	4
Finevision Influenza	4	4	4	4	5	5	3.5	5	5	5	4	4
RapidTesta FLU•NEO	4	4	4	4	5	5	3	5	4.5	4	4	4
Nanotrap Flu A•B	4	5	4.5	4	5	5	4	5	5	5	4.5	5
Quick Chaser Flu A,B (Type H)	3	4	3.5	3	4	4	3	5	4	4.5	4	4
Alsonic Flu	4	4	4	4	5	5	4	5	5	5	4	4
Primecheck Flu (Type S)	4	4	4	4	4	5	3	5	4	5	4	4
Primecheck Flu•RSV	4	4.5	5	4	5	5	4	5	5	5	4.5	4.5
BD Veritor System Flu	3	3.5	3	3	4	4	3	5	4.5	5	4	4
Fuji dri-chem immuno AG cartridge FluAB	3	3	3	3	3	4	3	4	3.5	3	3	3
Spotchem FLORA FluAB	3	3.5	3	3	4	4	3	4.5	4	4	4	4
Immunotrap Influenza A•B	5	6	5.5	5	5.5	6	5	6	6	6	5	5
Rapiim Flu-AB	3	3	3	3	4	4	4	5	5	5	4	4

^a^Ten-fold serial dilutions of the indicated viruses (10^1^–10^6^ TCID_50_ per 100 μl) were examined with each IRDT according to the manufacturers’ instructions. The minimum viral titers required for a positive reaction were determined in two independent experiments. The average titers are shown.

We next examined the sensitivity of the 25 IRDTs against H5N1, H5N2, and H5N6 viruses. These H5 viruses are circulating in avian species and have the potential to transmit to humans^15–19^. The detection limit of the tested IRDTs ranged from 10^2^ to 10^6^ TCID_50_ per 100 μl for H5N1 viruses and H5N2 viruses and from 10^4^ to 10^7^ TCID_50_ per 100 μl for H5N6 viruses (Table 4). This finding indicates that the sensitivity of the IRDTs for H5N6 viruses (H5-5, -6, and -7) was 10‒100 lower than that for H5N1 and H5N2 viruses (H5-1, -2, -3, and -4). The detection limits of each IRDT for H5N1 viruses were lower than those in our previous experiments^14,20^. For H7 viruses, we used highly pathogenic H7N9 isolates (H7-2 and -3) from humans that emerged in the 2016‒17 season in China^7,8^, and a prototype H7N9 virus (H7-1)^5^. H7N2 virus (H7-4), which caused an outbreak in cats^21^, was also examined. The detection limits of the tested IRDTs for these H7 viruses ranged from 10^3.5^ to 10^7^ TCID_50_ per 100 μl. All tested H7 isolates were detected by the IRDTs with varying sensitivity and the sensitivity was comparable to or slightly lower than that for type B viruses. The most sensitive IRDT for H5 and H7 viruses was Fuji dri-chem immuno AG cartridge FluAB.

**Table 4:** Sensitivity of IRDTs for H5 and H7 viruses^a^.

IRDT	Minimum Virus Titer (log_10_ TCID_50_/100 μl) for a positive result with										
	H5-1	H5-2	H5-3	H5-4	H5-5	H5-6	H5-7	H7-1	H7-2	H7-3	H7-4
Statmark FLU Stick-N	5	4.5	4	4	5.5	6	6	5	6	6	5
RapidTesta color FLU stick	4.5	4	3	4	5.5	6	6	5	5	5	5
QuickVue Rapid SP influ	5	4	3.5	4	5	6	6	5	5.5	5.5	5
Clearview Exact Influenza A&B	4	4	3	3	5	6	6	4.5	5	5	5
Espline Influenza A&B-N	4	3.5	3	3	5	5	5	4	4.5	5	4.5
ImmunoAce Flu	4	3	3	3	5	6	6	4	4.5	5	4.5
Brightpoc Flu	4	4	3	4	5	6	5.5	5	6	6	5
Immunofine FLU	4	4	3	4	5	6	6	5	5	5	5
Spotchem i-Line FluAB-S	5	4	3	4	5.5	6	6	5	5	6	5
QuickNavi Flu	4	4	3	3	5	5	5	4	5	5	5
QuickNavi-Flu+RSV	4	4	3	3	5	5.5	5	5	5	5	5
Goldsign FLU	4.5	4	3	4	5	6	6	5	5	5	5
Prorast Flu One	3	3	2	3	4	5	5	4	4	4	4
Finevision Influenza	5	5	3	4	6	6	6	5	5	6	5
RapidTesta FLU•NEO	4	4	3	3	5	6	6	4	5	5	5
Nanotrap Flu A•B	4	4	3	3.5	5	6	6	4.5	5	5	5
Quick Chaser Flu A,B (Type H)	4	3.5	3	3	5	6	6	4	5	5	5
Alsonic Flu	4	4	3	4	5	6	6	5	5	5	5
Primecheck Flu (Type S)	4	4	3	3	5	6	6	4	5	5	5
Primecheck Flu•RSV	4	4	3	4	5	6	6	5	5	5.5	5
BD Veritor System Flu	4	3	2.5	3	5	5	5	4	4	4.5	4
Fuji dri-chem immuno AG cartridge FluAB	3	3	2	2	4	5	4.5	3.5	3.5	4	3.5
Spotchem FLORA FluAB	4	3	2.5	3	5	5	5	4	4.5	5	4
Immunotrap Influenza A•B	6	6	5	4.5	7	7	7	6	7	7	6
Rapiim Flu-AB	3.5	3	2.5	3	4	5	5	4	4.5	4.5	4

^a^Ten-fold serial dilutions of the indicated viruses (10^1^–10^6^ TCID_50_ per 100 μl) were examined with each IRDT according to the manufacturers’ instructions. The minimum viral titers required for a positive reaction were determined in two independent experiments. The average titers are shown.

In this study, all tested IRDTs detected seasonal H1N1pdm09, H3N2, and type B viruses, as well as H5N1, H5N2, H5N6, H7N2, and H7N9 viruses, which are potentially transmittable to humans^7,8,15–19,21^. Most of the IRDTs tested in this study showed higher sensitivity for seasonal influenza viruses than did the IRDTs we tested previously^14^, indicating that the sensitivity of IRDTs has improved.

For H1N1pdm09, H3N2, and type B viruses, which are the main targets for all IRDTs, the sensitivity of the analyzer-based IRDTs was similar to or better than that of the conventional IRDTs. In the case of seasonal viruses, virus titers usually peak at 10^2^‒10^6^ TCID_50_ per 100 μl of nasopharyngeal wash during the first 24–72 h of illness^22^. Therefore, most IRDTs tested could accurately detect influenza virus in patients during this period. However, for H5 and H7 viruses, the analyzer-based IRDTs tended to show greater sensitivity than the conventional IRDTs. In particular, Fuji dri-chem immuno AG cartridge FluAB detected H5 and H7 viruses at a sensitivity level comparable to that for seasonal influenza A and B viruses; the detection limits were 10^2^‒10^5^ and 10^3^‒10^4^ TCID_50_ per 100 μl, respectively. IRDTs possessing high sensitivity for potentially zoonotic H5 and H7 viruses are thus available to diagnose influenza caused by such viruses.

## Materials and Methods

### Diagnostic tests

The IDRTs listed in Table 1 were purchased from the manufacturers and evaluated for reactivity and sensitivity according to the manufacturers′ procedures. Rapiim™ Flu-AB requires an analyzer to read the test results and only a rental analyzer was available. Test samples were adjusted to 10^1^ to 10^6^ TCID_50_ per 100 μl with Eagle’s minimal essential medium (EMEM) containing 0.3% bovine serum albumin (BSA). The minimum virus titres required for a positive reaction were determined in duplicate examinations. The average virus titre for a positive reaction of two examinations is shown in the tables.

### Viruses

The influenza viruses listed in Table 2 were propagated in MDCK cells or chicken embryonated eggs. Their virus titres (TCID_50_) were determined using MDCK cells.

### Biosafety statements

All experiments with H5N1, H5N2, H5N6, and H7N9 viruses were performed in biosafety level 3 (BSL3) laboratories at the University of Tokyo, which are approved for such use by the Ministry of Agriculture, Forestry, and Fisheries, Japan.

### Data availability

All data analyzed during this study are included in this published article.

## References

[1] Harfoot R, Webby RJ (2017). H5 influenza, a global update. Journal of microbiology.

[2] Yang ZF, Mok CK, Peiris JS, Zhong NS (2015). Human Infection with a Novel Avian Influenza A(H5N6) Virus. The New England journal of medicine.

[3] Pulit-Penaloza JA (2015). Pathogenesis and Transmission of Novel Highly Pathogenic Avian Influenza H5N2 and H5N8 Viruses in Ferrets and Mice. Journal of virology.

[4] Kaplan, B. S. *et al*. Novel Highly Pathogenic Avian A(H5N2) and A(H5N8) Influenza Viruses of Clade 2.3.4.4 from North America Have Limited Capacity for Replication and Transmission in Mammals. *mSphere* **1**, 10.1128/mSphere.00003-16 (2016). 10.1128/mSphere.00003-16 PMC4894690 27303732

[5] Gao R (2013). Human infection with a novel avian-origin influenza A (H7N9) virus. The New England journal of medicine.

[6] Su, S. *et al*. Epidemiology, Evolution, and Pathogenesis of H7N9 Influenza Viruses in Five Epidemic Waves since 2013 in China. *Trends in microbiology*, 10.1016/j.tim.2017.06.008 (2017). 10.1016/j.tim.2017.06.008 28734617

[7] Zhang F (2017). Human infections with recently-emerging highly pathogenic H7N9 avian influenza virus in China. The Journal of infection.

[8] Zhu, W. *et al*. Biological characterisation of the emerged highly pathogenic avian influenza (HPAI) A(H7N9) viruses in humans, in mainland China, 2016 to 2017. *Euro surveillance: bulletin Europeen sur les maladies transmissibles* = *European communicable disease bulletin* **22**, 10.2807/1560-7917.ES.2017.22.19.30533 (2017). 10.2807/1560-7917.ES.2017.22.19.30533 PMC5476987 28537546

[9] Yamashita M (2010). Laninamivir and its prodrug, CS-8958: long-acting neuraminidase inhibitors for the treatment of influenza. Antiviral chemistry & chemotherapy.

[10] McNicholl IR, McNicholl JJ (2001). Neuraminidase inhibitors: zanamivir and oseltamivir. The Annals of pharmacotherapy.

[11] Watanabe M, Nukuzuma S, Ito M, Ihara T (2011). Viral load and rapid diagnostic test in patients with pandemic H1N1 2009. Pediatrics international: official journal of the Japan Pediatric Society.

[12] Watanabe M, Nakagawa N, Ito M, Ihara T (2009). Sensitivity of rapid immunoassay for influenza A and B in the early phase of the disease. Pediatrics international: official journal of the Japan Pediatric Society.

[13] Richard M, Fouchier RA (2016). Influenza A virus transmission via respiratory aerosols or droplets as it relates to pandemic potential. FEMS microbiology reviews.

[14] Sakai-Tagawa Y (2014). Detection sensitivity of influenza rapid diagnostic tests. Microbiology and immunology.

[15] Shen YY (2016). Novel Reassortant Avian Influenza A(H5N6) Viruses in Humans, Guangdong, China, 2015. Emerging infectious diseases.

[16] Ogata T (2008). Human H5N2 avian influenza infection in Japan and the factors associated with high H5N2-neutralizing antibody titer. Journal of epidemiology.

[17] Wu HS (2014). Influenza A(H5N2) virus antibodies in humans after contact with infected poultry, Taiwan, 2012. Emerging infectious diseases.

[18] Arafa AS (2016). Risk assessment of recent Egyptian H5N1 influenza viruses. Scientific reports.

[19] Lai S (2016). Global epidemiology of avian influenza A H5N1 virus infection in humans, 1997-2015: a systematic review of individual case data. The Lancet. Infectious diseases.

[20] Sakai-Tagawa Y (2010). Sensitivity of influenza rapid diagnostic tests to H5N1 and 2009 pandemic H1N1 viruses. Journal of clinical microbiology.

[21] Belser, J. A. *et al*. A Novel A(H7N2) Influenza Virus Isolated from a Veterinarian Caring for Cats in a New York City Animal Shelter Causes Mild Disease and Transmits Poorly in the Ferret Model. *Journal of virology* **91**, 10.1128/JVI.00672-17 (2017). 10.1128/JVI.00672-17 PMC5512233 28515300

[22] World Health Organization Writing G (2006). Non-pharmaceutical interventions for pandemic influenza, international measures. Emerging infectious diseases.

